# Algorithmic recommendation and adolescent mental health: a grounded theory study of social and commercial determinants

**DOI:** 10.1186/s12889-026-28300-5

**Published:** 2026-06-24

**Authors:** Lizzy Winstone, James Parsonage, Lauren Cross, Cassey Muir, Amie Randhawa, Georgie MacArthur, Emma Garavini, Evangelina Asiedu-Addo

**Affiliations:** 1 Population Health Sciences, University of Bristol, Bristol, UK; 2 MRC Epidemiology Unit, University of Cambridge, Cambridge, UK; 3 Population Health Sciences Institute, Newcastle University, Newcastle, UK; 4 School of Sport, Exercise and Rehabilitation Sciences, University of Birmingham, Birmingham, UK; 5 The McPin Foundation Young People’s Network, The McPin Foundation, London, UK

**Keywords:** Algorithmic recommendation, Adolescent mental health, Social media, Grounded theory, Socioecological model, Commercial determinants of health

## Abstract

**Background:**

Existing digital mental health research tends to frame social media in individualistic terms, emphasising screen-time, self-control, or personal vulnerability. Less attention has been given to the role of algorithmic recommendation systems in population mental health. We aimed to develop a grounded theory of how adolescents experience algorithmically curated feeds and how these experiences relate to mental health and wellbeing.

**Methods:**

We conducted semi-structured, photo-elicitation interviews with 27 UK young people aged 14–19 years. Participants shared screenshots from their TikTok ‘For You’ and Instagram ‘Explore’ pages to support discussion of algorithmically recommended content. Data were analysed using constructivist grounded theory, with iterative coding, memo-writing, and theoretical sampling.

**Results:**

We identify algorithmically structured exposure as a central mechanism through which engagement-driven recommendation systems shape young people’s digital mental health. Algorithms organised what content participants encountered, how frequently, and with what emotional intensity, within broader socioecological contexts. Three interlocking processes sustained exposure; algorithmic reinforcement of engagement, emotional feedback loops between mood and recommendations, and a persistent sense of limited control despite high awareness. Although participants developed adaptive strategies and algorithmic literacy, these were unevenly distributed and often insufficient within engagement-driven, profit-oriented systems.

**Conclusions:**

Digital mental health may be produced through interactions between individual capacities, social environments, and commercially driven platform design. Protecting population mental health therefore requires multi-level action, including developmentally appropriate social media and algorithmic literacy education alongside structural reforms to recommendation systems, transparency, and platform governance.

**Supplementary Information:**

The online version contains supplementary material available at https://doi.org/10.1186/s12889-026-28300-5.

## Introduction

Recent increases in anxiety, depression, and self-harm among adolescents have intensified concern about the social and environmental determinants of youth mental health [1, 2]. Social media platforms now form a central part of adolescents’ everyday environments, shaping how young people communicate, access information, construct identities, and seek support [3–5]. In many countries, social media use is nearly universal during adolescence [4]. As a result, these platforms represent an increasingly important setting for population mental health and wellbeing.

Research indicates that social media affords connection, creativity, and peer support, while also exposing young people to social comparison, harassment, and distressing content [6, 7]. These experiences are socially patterned, varying by gender identity, socioeconomic position, ethnicity, sexuality, and prior mental health status [8–11]. Certain groups appear more likely to encounter harmful content or to experience negative emotional consequences, while others may benefit more from opportunities for community and expression [8–11]. Qualitative work with young people facing mental health difficulties similarly highlights that vulnerability is shaped by the interaction between personal circumstances and the risky affordances of platforms, rather than by individual characteristics alone [12]. From a public health perspective, this unevenness highlights the need to understand for whom and under what conditions social media affects mental health. Social media has recently been described explicitly as a determinant of health within public health literature, reflecting its pervasive influence on information environments, social relationships, health behaviours, and political discourse [13].

Much existing digital mental health research frames social media in individualistic terms, emphasising screen-time, self-control, or personal vulnerability, and often reducing complex digital experiences to single exposure metrics [3, 6, 14–16]. Although such approaches have generated valuable insights, they provide limited understanding of the processes through which social media environments operate in young people’s lives.

An increasingly recognised limitation of this literature is the comparatively limited attention paid to algorithmic recommendation systems and the ways they shape young people’s digital environments [12, 17]. Although some platforms retain optional chronological viewing modes, algorithmically curated recommendation systems now dominate content visibility across major social media environments, including TikTok’s “For You” feed and Instagram’s default “Home” and “Explore” feeds. Recommendation systems may operate differently across digital contexts and affordances, including discovery feeds, follower-based streams, and messaging environments [18–20]. In highly curated discovery feeds such as TikTok “For You” and Instagram “Explore” (the focus of our study), algorithms prioritise and repeat material based on users’ behaviour and predicted engagement. These systems determine what young people see, how often they see it, and in what combinations. Algorithmic recommendation systems therefore structure exposure over time, shaping the digital environments that different individuals and groups encounter, and potentially influencing population mental health [5, 21–23].

Although algorithmic systems are increasingly recognised within digital media and communication literature, they remain comparatively under-integrated within adolescent mental health and public health frameworks. There is a particular lack of research centring young people’s lived experiences of cumulative algorithmically curated exposure within everyday mental health and wellbeing contexts. Qualitative studies are essential to capture how repeated exposure unfolds over time, how young people interpret and negotiate recommended content, or how meanings and emotional responses shift across contexts. Understanding these processes requires approaches capable of exploring agency, perception, and lived experience in depth [21, 22]. Existing audit, computational, and quantitative research has provided important insights into platform dynamics and algorithmic amplification [20, 24–26], but less is known about how adolescents themselves experience, interpret, and attempt to manage cumulative algorithmically curated exposure within everyday mental health and wellbeing contexts.

Using constructivist grounded theory [27], we explored how young people experience and interpret algorithmically curated social media on TikTok “ForYou” and Instagram “Explore”, in relation to mental health and wellbeing. By focusing on highly curated discovery feeds and using constructivist grounded theory [27] with photo-elicitation interviews [28, 29], this study aimed to develop a socioecological account of how recommendation systems shape young people’s digital mental health experiences within commercially driven online environments. Rather than asking whether social media is harmful in general, we sought to identify the processes through which engagement-driven platforms shape emotional trajectories and everyday experience. Such understanding is essential for informing policies that address not only individual skills, but also the structural conditions that pattern digital mental health.

## Methods

### Study design and participants

Participants were recruited using multiple pathways, including the McPin Foundation’s Young People’s Network, outreach to Personal, social, health and economic education (PSHE) leads or headteachers in secondary schools across the UK, and contact with youth mental health and youth organisations. Study materials and procedures were developed in collaboration with lived experience experts from the #ForYou Young People’s Advisory Group (McPin Foundation, *N* = 6) to ensure relevance, accessibility, and ethical sensitivity.

Twenty-seven young people aged 14–19 years took part (mean age 17.15 years, SD 1.49). Nineteen participants identified as female, seven as male, and one as gender diverse. Recruitment strategies aimed to include a range of mental health experiences, but mental health history and other demographic characteristics were not systematically recorded.

### Data collection

Prior to interview, participants attended a brief (approximately 15-minute) online meeting in which the researcher introduced themselves, explained the study, addressed any questions and safety considerations, and arranged the interview. Participants shared 2–10 screenshots from their TikTok “For You” or Instagram “Explore” pages prior to interview, balancing flexibility and minimising participant burden. Instagram screenshots often contained multiple pieces of recommended content, meaning fewer screenshots were typically needed to elicit a range of material for discussion. Screenshots could represent typical recommended content or material that felt particularly salient or meaningful to participants. Photo-elicitation [29] enabled discussion of concrete, recent algorithmically recommended content, enhancing ecological validity. Visual elicitation approaches may be particularly valuable in research involving sensitive topics or younger populations by grounding discussion in familiar experiences and supporting participant reflection and engagement [28]. Through these methods, we aimed to make algorithmic recommendation systems less abstract by grounding interviews in participants’ everyday social media experiences. Screenshots were used as prompts (rather than objects of analysis themselves) to support richer reflection on real examples of recommended content, facilitating discussion of how young people interpreted and experienced algorithmic curation [28, 29]. Interviews focused primarily on participants’ experiences of algorithmically recommended discovery feeds, specifically TikTok “For You” and Instagram “Explore”, as these environments were understood to involve intensive and repeated forms of personalised content exposure.

A topic guide was co-developed with the #ForYou Young People’s Advisory Group (McPin Foundation, *N* = 6) (see supplementary file), was used flexibly, and updated iteratively. Topics covered general social media use, perceptions of algorithms, positive and negative experiences of algorithmically recommended content, and social media literacy, as well as questions about the specific content shared in advance. Interviews lasted approximately one hour, were audio-recorded with consent, and transcribed verbatim.

### Researchers and reflexivity

Interviews were conducted between March and September 2025 by LW (female, PhD), an experienced qualitative researcher, and JP (male, MSc), who had received training but was new to qualitative interviewing. Reflexive memos were written after each interview to document analytic insights, emerging questions, and the researchers’ positionality in relation to the data.

### Data analysis

Analysis followed a constructivist grounded theory approach [27, 30]. Open and focused coding were conducted iteratively, supported by memo-writing and constant comparison. Theoretical sampling informed category refinement. Team discussions and youth advisory input were used to support analytic rigour. Data were managed using NVivo (version 14).

### Ethics and consent

The study was conducted in accordance with the Declaration of Helsinki and ethical approval was granted by the University of Bristol Faculty of Health Sciences Research Ethics Committee (ref: 22479). All participants provided informed consent prior to taking part. For participants aged under 16 years, informed parental or guardian consent was obtained in addition to informed participant assent. Following the interview, participants were provided with information about relevant support services and tips to safely manage their social media feeds.

## Results

**Fig. 1:**
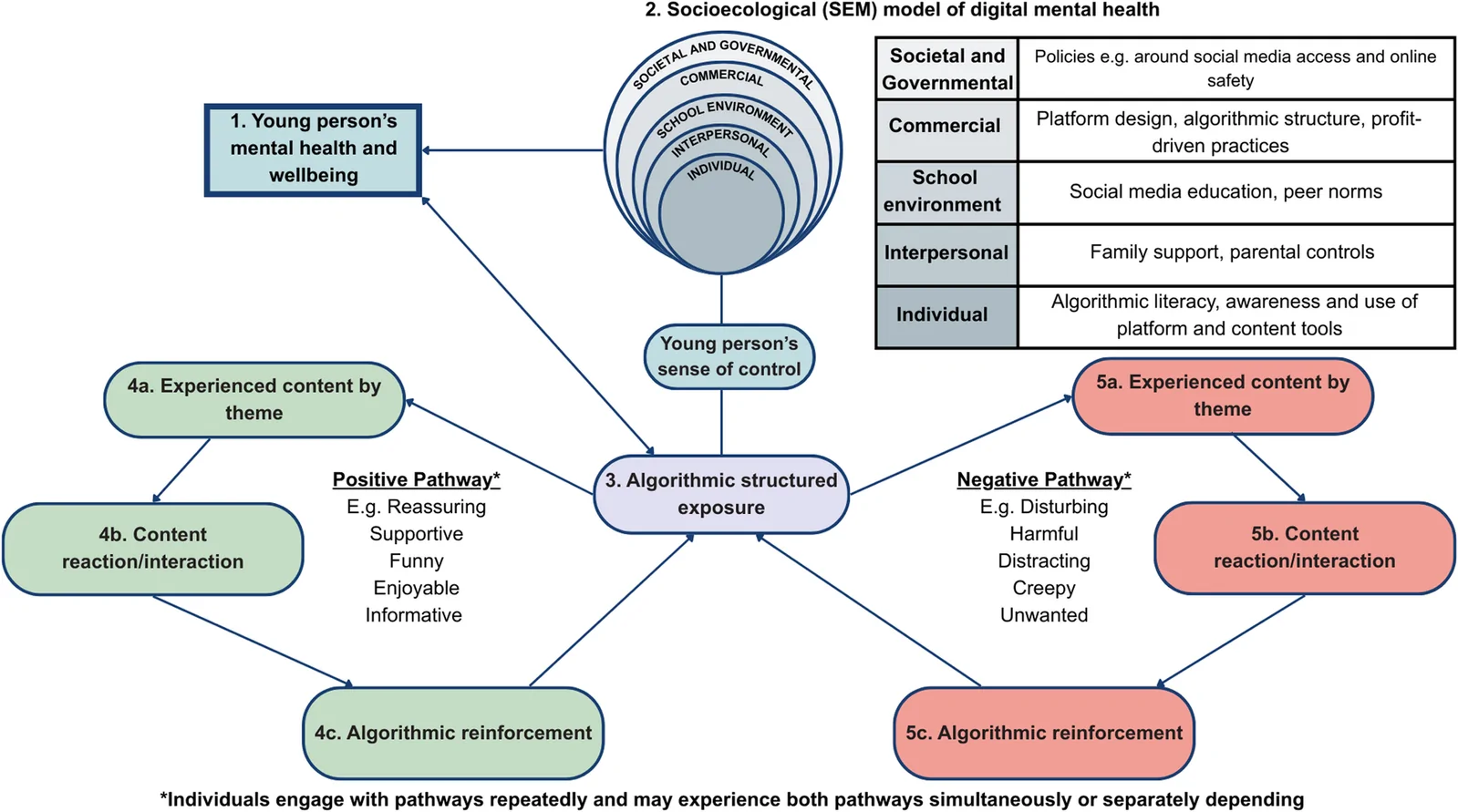
Grounded theory model of algorithmically structured exposure within a socioecological framework

We propose a socioecological model of algorithmically shaped digital mental health (Fig. 1). The core category generated through grounded theory analysis was algorithmically structured exposure, capturing how engagement-driven recommendation systems organise cumulative patterns of content visibility over time. This process comprised three interlocking subcategories: i) algorithmic reinforcement, ii) emotional feedback loops, and iii) perceived lack of control and the limits of adaptive strategies (Table 1).

**Table 1:** Mapping of applied codes to analytic categories

Results category	Sub-category	Illustrative codes
Core category: Algorithmically structured exposure within socioecological contexts	Individual-level influences	Feed reflects MH; Scroll more when depressed; Staying at home more vulnerable; Self-reflection; Comparison; Distraction from problems; Health anxiety; Self-esteem affects resilience
	Interpersonal contexts	Advice from family members; Parent SML important; Peer activity shapes algorithm; More likely to share funny content with friends; Community in comments
	Structural influences	Business models; Attention online via engagement; Exploitative; Controversy or shock leads to more engagement; Algorithm privacy concerns
	Definitions of healthy use	Purposeful SMU healthy; Balance in life supports SML and resilience; Needs mindful effort; Communication healthier than feed
Key processes underpinning algorithmically structured exposure	Algorithmic reinforcement	Liking content shapes feed; Searches to tailor feed; Random suggestions; Trending topics; Algorithm understanding; Demographic information shaping feed
	Emotional feedback loops	Algorithm-driven spirals; Doom-scrolling; Hover too long; Scroll more when depressed; Political topics distressing; Night-time exposure
	Perceived lack of control and the limits of adaptive strategies	Difficult to avoid app; Difficult to look away; Addictive; Lose track of time; Waste of time; Brain rot; Algorithm difficult to escape; Block accounts; Not interested button; Scroll past; Delete app; Detox; Breaks; Tailoring feed; SML through experience; Younger users vulnerable; School SML insufficient; Need for education reform
Mechanisms of influence on digital mental health	Content types – entertainment	Funny animal content; Relaxing; Living vicariously; Positive reinforcement; TikTok became less fun
	Content types – mental health	Validating content; Triggering content; Recovery content gateway; Misuse of MH diagnoses
	Content types – identity	LGBTQ+ community; Autism; Representation; Community in comments; Discrimination; Hashtag misclassification
	Content types – body image	Unrealistic beauty standards; Body positivity; Beauty drives engagement; Resistant to SML/MHL; Objectification
	Content types – consumerism	Adverts; Influencer culture; Exaggeration; Commercial pressure
	Content types – news/activism	Political echo chambers; Guilt-inducing exposure; Watching as activism; Moral overload
	Content types – AI-generated	Disturbing AI content; Uncanny imagery; Misinformation; Mistrust
Perceived lack of control and the limits of adaptive strategies	Commodifying platform design	Business models; Attention as resource; Engagementmonetised; Privacy concerns; Sensationalised conflict; Exploiting others for content; Misogyny; Shock content; Content moderation; Algorithm protective/restrictive; Age restrictions' So the 'results category' column should only have three cells - 'Core category: Algorithmically structured exposure within socioecological contexts', 'Key processes underpinning algorithmically structured exposure' and 'Mechanisms of influence on digital mental health
		Business models; Attention as resource; Engagement monetised; Privacy concerns; Sensationalised conflict; Exploiting others for content; Misogyny; Shock content; Content moderation; Algorithm protective/restrictive; Age restrictions

### Core category: Algorithmically structured exposure within socioecological contexts

Young people described social media experiences as shaped less by individual posts than by repeated, patterned exposure produced by algorithmic recommendation systems. Mental health and wellbeing were influenced by how content accumulated and recurred over time, interacting dynamically with mood, identity, peer relationships, and offline environments such as school and home. Algorithms therefore shaped not only what was seen, but how often, in what combinations, and under what emotional conditions.

These experiences were embedded within a wider socioecological system. At the individual level, digital mental health appeared linked to emotional state, developmental stage, and mental health literacy. At the interpersonal level, peer norms, family attitudes, and school cultures seemed to shape expectations around social media use and strategies for responding to content. At the structural level, platform design, recommendation systems, and commercial incentives were understood to prioritise engagement over wellbeing. Although broader policy influences were less explicitly discussed, participants frequently framed their experiences as constrained by business models and the absence of meaningful regulation.

Young people articulated their own definitions of healthy social media use. These emphasised i) regular exposure to positive or supportive content; ii) the ability to disengage from material that elicited negative emotional responses; iii) moderating screen-time balanced with offline activities; iv) critical awareness of self-presentation and algorithmic processes; and v) use that is oriented toward direct communication with peers rather than passive scrolling. However, achieving these goals was reportedly undermined by algorithmic dynamics beyond their control, positioning digital wellbeing as a product of interacting socioecological influences rather than individual behaviour alone.

### Key processes underpinning algorithmically structured exposure

The following interlocking processes describe how commercial recommendation systems may generate and sustain algorithmically structured exposure. Participants demonstrated varying forms of algorithmic awareness. Many described a practical understanding of how engagement shaped recommendations, developed through everyday platform use, experimentation, and trial-and-error experiences of managing their feeds. Some participants appeared to have stronger understanding of algorithmic systems, for example through school-based learning opportunities, prior content creation experience, or support from technologically knowledgeable family members. Others demonstrated more limited understanding and relied primarily on peers or experiential learning to interpret how recommendation systems operated. Despite differences in technical knowledge, most participants described developing adaptive strategies to manage their feeds and protect their wellbeing, including limiting engagement with distressing content, using platform tools such as ‘not interested’, and consciously reshaping recommendation patterns over time.

#### Algorithmic reinforcement

Algorithmic reinforcement was determined to be a central mechanism shaping patterned exposure. Participants described interaction, including likes, comments, follows, shares, and viewing behaviour, as the primary way platforms learned about them. These interactions were understood to function as training data, progressively shaping future recommendations. As engagement accumulated, content became increasingly organised around inferred interests, identities, or emotional states rather than appearing incidentally or by choice.

*“It’s some form of mathematical calculation… so it skews the types of videos you’re going to see… to try and keep you engaged for longer.” (Gender diverse*,* 17)*.

Reinforcement frequently occurred without deliberate intent. Engagement driven by curiosity, concern, disagreement, or attempts to support others was still registered as interest, resulting in further amplification of similar material. Exposure was therefore shaped not only by what young people wanted to see, but by how platforms interpreted attention within systems designed to maximise engagement. Many participants framed this process as exploitative, describing how platforms were perceived to capitalise on emotion and reaction to extract further attention and data, regardless of users’ mental health and wellbeing.

#### Emotional feedback loops

Emotional feedback loops further shaped exposure by linking mood, engagement, and content visibility over time. Participants described cyclical relationships between mood, engagement, and subsequent recommendations. In some cases, this produced spirals in which emotionally congruent content became increasingly prominent and difficult to escape.

*“If you’re not feeling so great… and you go on Instagram for a bit of a break you happen to hesitate a couple of times on some videos that are*,* kind of*,* reflective of how you’re feeling at the minute*,* and then it just spirals.” (Female*,* 19)*.

These loops operated in both positive and negative directions. Comforting content could reinforce belonging, while distressing material could intensify low mood or comparison. Some participants noted that algorithms did not appear responsive to shifts in need; content linked to past vulnerabilities often resurfaced even after circumstances had changed. This persistence contributed to feelings of being “stuck” within particular exposure trajectories.

#### Perceived lack of control and the limits of adaptive strategies

Despite high levels of algorithmic awareness, participants frequently described a persistent sense of limited control. Altering recommendation patterns (through use of platform tools such as “not interested,” blocking, or reporting) was experienced as slow, effortful, and inconsistent, with previous interactions exerting a lingering influence.

*“Those involuntary actions… are all taken down and used to influence that. So*,* I don’t really get a choice in the matter of whether it’s going to suggest more of that content to me or not.” (Female*,* 17)*.

When strategies felt insufficient, some withdrew from platforms, though this was often socially costly, limiting participation in peer conversations and shared cultural references relating to social media. Even deliberate attempts to temporarily disengage were often undermined by platform design.

*“Just when you’re about to click off… you get sucked back into the cycle.” (Female*,* 16)*.

Participants also expressed discomfort at how accurately platforms appeared to infer their interests or emotional states, describing this as intrusive or “creepy”. A recurring tension emerged between intentions and algorithmic outcomes, whereby actions taken to challenge content, seek information, or support others resulted in further exposure to unwanted material.

*“TikTok doesn’t know that I’m commenting because I dislike it. So it brings me more.” (Male*,* 18)*.

At the same time, young people described developing forms of social media and algorithmic literacy through lived experience rather than formal education. Recognising recommendation patterns, identifying emotionally triggering content, and using platform tools were widely viewed as protective strategies. However, these adaptive practices were socially patterned and structurally constrained. Participants described uneven access to support from parents, schools, and other trusted adults in understanding and managing algorithmically recommended content. Some young people reported receiving guidance from adults who were perceived to be digitally engaged, or attending school sessions involving technology companies that discussed algorithms and online safety, while others described little formal education beyond generic warnings about screen-time or social media use. Peer norms and family resources also shaped opportunities to manage content. Some participants described barriers linked to intergenerational, linguistic, or cultural differences in social media use and understanding.

*“I spoke to my mum… she can’t really relate*,* she doesn’t she doesn’t really understand it. I didn’t really find it helpful.” (Female*,* 17)*.

School-based provision was experienced as inconsistent in both quality and specificity, with many participants feeling that education focused primarily on generic online safety rather than recommendation systems, emotional reinforcement, or mental health.

*“There needs to be better education than just ‘It’s bad*,* don’t use it*,*’ because that’s not realistic.” (Female*,* 19)*.

Although recognising emotionally harmful patterns and using protective strategies (such as limiting engagement or using platform tools) were often viewed as helpful, participants frequently felt these efforts were insufficient within engagement-driven platforms designed to sustain attention and repeated exposure. These accounts illustrate that perceived loss of control was not simply a matter of individual self-regulation. Rather, young people’s capacity to manage their digital environments was shaped by interacting influences across socioecological levels, while algorithmically structured exposure continued to influence digital mental health in ways that could not be fully mitigated by awareness and adaptive effort alone.

### Mechanisms of influence on digital mental health

#### Content types as expressions of algorithmically structured exposure

These processes operated across diverse recommended content types, including entertainment, mental health narratives, identity-related material, body image content, consumerism, news, and AI-generated posts. Regardless of topic, reinforcement and feedback loops shaped the clustering, intensity, and emotional effects of repeated exposure. Harm and benefit were therefore less dependent on content category than on cumulative exposure patterns and engagement-driven amplification.

#### Commodifying platform design

Participants consistently appeared to interpret recommendation systems as fundamentally commercial. Engagement, whether positive, negative, or accidental, seemed understood to be converted into reach and revenue.

*“It doesn’t matter if you’re giving it negative attention*,* it’s still attention which will see the video go further and make the platform money.” (Male*,* 16)*.

Participants also noted the pervasive presence of advertising and branded content within feeds, interpreting recommendation systems as mechanisms that maximise young users’ attention and exposure to commercial messaging. Exploitation was perceived both in the harvesting of users’ data and in content production practices that profited from conflict, distress, or the lives of vulnerable individuals.

*“Those types of videos unfortunately get a lot of engagement because it causes a reaction. And so I guess that’s more money for TikTok.” (Female*,* 19)*.

Such perceptions located algorithmically structured exposure within extractive digital economies, where emotions, relationships, and vulnerabilities were leveraged for commercial gain. Responsibility for harm was therefore attributed largely to platform design choices and commercial incentive structures rather than to individual users.

Within this model, young people’s experiences are produced through interactions between commercial platform design, peer norms, family and school contexts, algorithmic and mental health literacy, and individual coping strategies. Participants frequently described algorithms as shaping exposure in ways linked to platform commercial incentives. Although participants demonstrated substantial awareness and self-regulatory effort, these were often described as insufficient to prevent harm, highlighting how responsibility for safe use feels displaced onto individuals despite structural constraints.

## Discussion

These findings generate a grounded theory in which algorithmically structured exposure operates as a mechanism through which commercially driven platforms shape young people’s mental health, systematically influencing attention, emotional salience, and visibility in ways that intersect with adolescents’ developmental vulnerabilities and psychosocial environments. Digital mental health and wellbeing was shaped by the interaction of platform systems, social environments, and individual capacities, illustrating why awareness and personal strategies, while important, were rarely sufficient on their own to counteract the structural dynamics of algorithmic recommendation on discovery feeds.

This study advances existing research on adolescent social media and mental health by conceptualising algorithmically structured exposure as a lived and cumulative process operating across socioecological levels, rather than as isolated encounters with individual pieces of content. By centring young people’s experiences of highly curated discovery feeds, the findings extend existing work on digital harms and platform literacy through a public health and commercial determinants perspective. This reframing shifts the focus from individualised risk narratives toward the social and commercial conditions under which digital mental health and wellbeing is produced, consistent with calls to consider digital environments as upstream determinants of youth mental health [31]. This also aligns with arguments that social media platforms operate as commercial determinants of health, shaping what users encounter and how often, through profit-oriented design and recommendation systems rather than neutral content delivery [13, 23]. Emerging audit research further suggests that recommendation systems may be capable of inferring characteristics such as age and indicators of vulnerability from behavioural patterns [25], raising important questions about platform responsibilities where systems appear capable of identifying vulnerability while continuing to prioritise engagement-driven content delivery.

The socioecological model developed in this study (Fig. 1) resonates with emerging systems-based approaches to adolescent mental wellbeing, including causal loop models that conceptualise digital environments as part of wider interacting wellbeing systems [24]. Our findings build on this by providing a more detailed account of how algorithmically curated recommendation systems may shape young people’s lived experiences within highly personalised digital environments, and in relation to other socioecological systems. In particular, participants described repeated recommendation patterns as interacting dynamically with emotional states, peer norms, social media literacy, social supports, and existing vulnerabilities over time, suggesting that recommendation systems may operate as reinforcing mechanisms within broader digital wellbeing processes. Our model also highlights that positive and negative pathways may operate simultaneously. For example, emotionally validating mental health content may foster connection and reassurance while also reinforcing prolonged engagement with distress-related material. Understanding these overlapping processes may help future research move beyond simplistic framings of social media use as either harmful or beneficial. Consistent with the Youth Digital Skills (ySKILLS) framework [32], participants’ digital skills and algorithmic awareness shaped how they reportedly navigated online risks and opportunities. ySKILLS conceptualises digital skills as mediating relationships between opportunities, risks, and wellbeing, while emphasising that these processes are embedded within social, institutional, and platform contexts [32, 33]. Our findings extend this perspective by demonstrating that algorithmic literacy operates within structurally constrained and commercially driven environments, echoing recent research that shows young people with high levels of awareness still struggle to counteract engagement-driven exposure patterns [12, 33]. Even when participants showed high levels of competence, engagement-based recommendation systems frequently limited the protective value of individual skills. Moreover, some ostensibly protective strategies (such as withdrawing from platforms or reducing engagement) were described as carrying social costs. The effectiveness of individual competence therefore depends not only on algorithmic and commercial conditions, but also on the social contexts in which social media use is embedded [23].

Policy debates and public discussions often attribute harm to problematic use, while paying less attention to the mechanisms through which platforms organise attention and visibility [34]. As a result, responsibility for managing digital mental health is frequently located with individuals, e.g., through social media literacy, self-regulation, or parental mediation [3, 35]. However, evidence indicates that even highly aware and reflexive users can struggle to manage cumulative, personalised exposure within engagement-driven systems [12]. Equivalent attention to the structural conditions that shape online experiences is lacking [5]. This focus risks obscuring how algorithmic systems may reproduce or intensify existing social inequalities in exposure and impact.

A recent Lancet Psychiatry Commission [1] positions digital environments as upstream determinants of youth mental health but notes limited empirical evidence on how platform systems shape everyday experiences of wellbeing. Our findings address this gap by identifying algorithmically structured exposure as a central mechanism through which engagement-driven business models become embedded in adolescents’ socioecological contexts. Although responsibility for managing content-related experiences was frequently individualised, agency was structurally constrained by recommendation systems optimised for engagement. Our findings suggest that public mental health strategies must move beyond individual skill-building to address the commercial incentives and design features that systematically organise exposure [21]. Enhancing social media and algorithmic literacy remains essential, but is unlikely to be sufficient in isolation [4, 5, 29].

### Policy and intervention implications

The findings have timely relevance for debates around age-based social media restrictions. From a socioecological perspective, blanket bans risk treating social media as a uniform risk and young people as passive recipients, while leaving structural drivers of harm unchanged [23]. Instead, our results support systems-level reform targeting business models and platform design, including greater algorithmic transparency, limits on engagement-driven amplification, stronger moderation standards, and safer default settings for younger users [5, 23, 36]. Participants’ accounts suggest that literacy may be most protective when introduced early and supported institutionally, reinforcing the need for developmentally appropriate, curriculum-embedded social media and mental health education.

These implications align with wider public health recommendations that digital harms should be addressed primarily through structural and preventive action rather than through individual behaviour change alone. Public health approaches to digital determinants emphasise delaying harmful exposures, reducing risks embedded within platform design, and mitigating adverse consequences through regulation and institutional supports, rather than relying on young people’s self-regulation or parental mediation [5, 35]. Consistent with this perspective, our findings indicate that while individual skills and awareness are critical, they are routinely constrained by engagement-driven systems that shape exposure beyond users’ control. Individual-level behaviour change interventions are therefore necessary but insufficient in isolation [3, 37]. The socioecological model developed in this study suggests that future research and intervention efforts should examine how repeated recommendation patterns interact with emotional states, peer norms, school and family support, and platform design over time, in addition to exposure to specific forms of content.

A comprehensive public health response should be multi-level. First, at the individual level, young people require practical support to recognise when content becomes psychologically detrimental and to use platform tools effectively to manage their feeds. Second, at the interpersonal level, parents and carers may also require support in developing their own social media and algorithmic literacy in order to offer guidance and make effective use of available parental controls. Third, at the institutional level, early and developmentally appropriate social media and mental health literacy should be embedded within school curricula, with explicit attention to algorithms, emotional reinforcement processes, and commercial intent. Fourth, at the structural level, upstream action is required to address the design features that generate harm, including regulation of content recommendation systems, limits on engagement-driven amplification of harmful content, and enforceable transparency and accountability requirements for platforms. Shifting responsibility from individuals to the systems that organise digital exposure is essential to protecting adolescent mental health.

### Strengths and limitations

Strengths include in-depth qualitative data grounded in young people’s lived experiences and the use of photo-elicitation to enhance ecological validity and data richness [28, 29]. Use of theoretical sampling strengthened the analytic power of our conceptual categories within the grounded theory [30]. Limitations should be noted. The sample was UK-based and self-selecting, and we did not formally capture mental health status, ethnicity, or sexual orientation. This study focused primarily on highly algorithmically curated discovery feeds (TikTok “For You” and Instagram “Explore” pages), and findings may not transfer directly to other social media contexts such as messaging environments or follower-based feeds, where social dynamics, affordances, and recommendation processes may differ [19, 20]. Content creation was not explicitly explored. As with all qualitative research, findings offer transferable conceptual insights rather than generalisability.

## Conclusion

This study demonstrates that young people’s digital mental health and wellbeing is shaped primarily by cumulative, algorithmically structured exposure rather than by isolated pieces of content. Algorithmic recommendation systems operate as mechanisms through which commercially driven platforms shape digital mental health, organising attention, emotion, and visibility in ways that interact with developmental vulnerabilities and socioecological contexts. Although young people showed considerable awareness and skill in managing their online experiences, these individual strategies were often insufficient to counteract engagement-driven systems designed to maximise time on platform. Protecting mental health therefore requires shifting responsibility away from individual users toward structural change. A public health response should combine early, developmentally appropriate social media and algorithmic literacy education with upstream regulation of recommendation systems, transparency requirements, and stronger protections for younger users. By framing algorithmic influence as a modifiable structural driver of mental health, this study highlights the need for coordinated action that reshapes digital environments to better support young people’s wellbeing.

## Supplementary information

Supplementary Material 1.

## Data Availability

The data from this study will be available under Restricted access conditions from the University of Bristol data.bris repository.
