# Supplementary material for: Algorithmic recommendation and adolescent mental health: a grounded theory study of social and commercial determinants
Source: BMC Public Health. 2026 Jun 24;26:2526. doi: 10.1186/s12889-026-28300-5 (PMC13540923; doi:10.1186/s12889-026-28300-5)
Supplement: Supplementary file 1 [file 12889_2026_28300_MOESM1_ESM.docx]

**Algorithmic recommendation and adolescent mental health: a grounded theory study of social and commercial determinants**

**Supplementary material**

**Topic guide**

**Background**

- First, could you describe in your own words your relationship with social media

**[prompts if necessary]**

- How old were you when you started using [TikTok/ Instagram]?
- Has your social media use changed since you started? How?
- How often do you use [TikTok/ Instagram]? How much time each day do you spend on [TikTok/ Instagram]?
- How do you feel after you use it?
- How have the platforms changed since you started using social media?
- What sort of content to you look at on [TikTok/ Instagram]?
- Do you search for specific content or look at your Explore page or TikTok feed to see suggested content?
- Do you feel that your feed reflects your interests?
- If you hear the word ‘social media algorithm’, what does that make you think of? Could you explain what that means in your own words? How do you feel about social media algorithms?

**Photo discussion**

- Confirm platform for screenshots [TikTok/Instagram]
- What do you like about [TikTok/ Instagram]? What do you dislike about it?
- [SHARE screenshot(s)] Ask them to describe one or more screenshots of their *feed/ explore* page.
  - What kind of content are you seeing here?
  - Why do you think this content appeared on your feed?
  - Do you think this represents your typical *feed/Explore* page, or is this unusual?
- For each screenshot or specific posts:
  - Tell me about this image – is it a video? If so, what does the rest of the video show?
  - What made you choose this to bring to the interview? Did you engage with this image (like, comment, share, etc)?
  - How does this content make you feel (e.g., happy, anxious, entertained)?
  - What message do you think this content is showing? What was the person trying to achieve by sharing it?
  - Do you feel this kind of content affects your mental health in any way?
  - Do you think seeing this kind of content repeatedly affects how you think about yourself or the world?
  - Why do you think [TikTok or Instagram] decided to show you this content?
- Do you feel like you have control over what appears on your feed?
- Are there times when the content feels unrelated to what you’re interested in?
- What does your feed say about you?
- Do you notice your feed changing depending on how you’re feeling/the headspace you’re in at any one time?
- [Why do you think some people only get positive content suggested to them and other people see things that aren’t very nice?]

**Positive Impacts:**

- Are there things you like about how your feed is tailored?
- Have you discovered helpful or inspiring content through the algorithm?

**Negative Impacts:**

- Have you noticed content that made you feel uncomfortable, stressed, or self-conscious?
- Does your feed ever feel repetitive or overwhelming?
- How do you respond to negative content, e.g.,
  - What do you do if you see something that makes you feel bad? Would you talk to anyone about it?
  - Have you ever muted accounts, reported posts, or adjusted settings to change your feed, e.g., pressed the ‘don’t show me this kind of content’ button?

**Literacy/Strategies:**

- How have you learnt about social media content and how to avoid things that make you feel bad? Prompt: Experience? Friends? Family? School?
- Is this ever talked about at school? Is this helpful?
- **How would you define healthy vs unhealthy social media use?**
  - Would you describe your social media use as healthy or unhealthy? [Do you think this has changed over time?]
  - What do you think influences whether someone uses social media positively or negatively, [e.g. headspace, gender, sexual orientation, mh, peers, school, social media literacy, interests….]

**Recommendations:**

- If you could change how TikTok or Instagram selects content for your feed, what would you suggest?
- Do you think these platforms should do more to support mental health? How?

**Feedback:**

- What advice would you give to other young people about managing their *feed* and mental health?
- Is there anything else you’d like to share about social media, algorithms, or mental health?
- [Do you think it would be helpful to talk more about social media content with teachers/mental health professionals?]
